# Discrimination between regional biotypes of *Impatiens glandulifera* using a simple MALDI-TOF MS-based method for use with seeds

**DOI:** 10.1186/s13007-019-0412-1

**Published:** 2019-03-14

**Authors:** Michael A. Reeve, Kathryn M. Pollard

**Affiliations:** CABI, Bakeham Lane, Egham, Surrey TW20 9TY UK

**Keywords:** Matrix-assisted laser-desorption and ionisation time-of-flight mass spectroscopy, Himalayan balsam, Plant-biotype discrimination, Seed-protein analysis

## Abstract

**Background:**

We have recently developed a simple, rapid, and relatively-cheap method for matrix-assisted laser-desorption and ionisation time-of-flight mass spectroscopy (MALDI-TOF MS) sample preparation that is applicable to plant material (in addition to microbial and insect material), and have used this to discriminate between closely-related *Impatiens* species and between regional biotypes of the invasive weed *Impatiens glandulifera* (commonly known as Himalayan balsam) using leaf samples. In the current paper, we have developed a complementary MALDI-TOF MS-based method for use with seeds. We have employed a combination of principal-component analysis and blind-tested comparison between reference-sample MALDI-TOF MS spectra and test-sample spectra to discriminate, on the basis of the acid-soluble seed-protein spectra generated by our method, between four regional biotypes of *I. glandulifera* from within the UK that differ in their susceptibility to the biological control agent Himalayan balsam rust (*Puccinia komarovii* var. *glanduliferae*).

**Results:**

Peak-rich and highly-reproducible spectra were obtained and, in blind testing with test seeds collected in 2017 against reference seeds collected in 2017, we observed 100% identification accuracy in 12 blind tests. In blind testing with test seeds collected in 2016 against reference seeds collected in 2017, we observed 92% identification accuracy in 12 blind tests.

**Conclusions:**

MALDI-TOF MS analysis of seed material is able to discriminate between regional biotypes of *I. glandulifera*. MALDI-TOF MS therefore has the potential to improve the efficiency and efficacy of weed biological control using co-evolved natural enemies of invasive non-native plant species, through the matching of biological control agents with susceptible regional biotypes.

**Electronic supplementary material:**

The online version of this article (10.1186/s13007-019-0412-1) contains supplementary material, which is available to authorized users.

## Background

Originally from the foothills of the Himalayas, the annual plant *Impatiens glandulifera* (Balsaminaceae), commonly known as Himalayan balsam, was introduced as a garden ornamental into Kew Gardens, UK in 1839 [1]. *Impatiens glandulifera* has since naturalised, and has spread extensively throughout the UK to become one of the country’s most-prevalent invasive species. *I. glandulifera* can form dense monocultures, which decrease biodiversity and can have a negative impact on whole ecosystems [2–6]. In response, CABI initiated a biological control programme against *I. glandulifera* in 2006, and conducted surveys for natural enemies throughout the weed’s native range [7]. Consequent to these surveys, a rust fungus, identified as *Puccinia komarovii* var. *glanduliferae* [8], was prioritised for further assessment as a potential biological control agent due to its prevalence and because of the high level of damage it inflicts on *I. glandulifera* in the field. As part of this biological control programme, extensive studies were carried out using a strain of the rust (IMI 398718) from Kullu Valley, Himachal Pradesh, India. These studies were conducted under quarantine conditions, and they elucidated the life-cycle, infection parameters, and host-range for this rust strain. The host-range studies assessed the susceptibility of 74 plant species to the rust, and the test-plant list included native, ornamental, and economically-important European plant species [9, 10]. From these studies, the rust *P. komarovii* var. *glanduliferae* was found to be highly host-specific.

The above research programme was incorporated into a Pest Risk Assessment (PRA), and the Indian strain of the rust was released in England in September 2014 [11] after governmental approval. Studies of field releases of the Indian rust strain across England and Wales in 2015 and 2016 revealed that regional biotypes of *I. glandulifera* differ in their susceptibility towards this particular biological control agent, with some regional biotypes being fully susceptible, and others resistant [12]. This, combined with results reported by Nagy and Korpelainen [13], who found that *I. glandulifera* was introduced into the UK multiple times from different locations within the native range (from both India and Pakistan), strongly suggests that multiple regional biotypes of *I. glandulifera* exist within the UK. In response to this complexity, a second strain of the rust (IMI 505791), originating from Kaghan Valley, Khyber Pakhtunkhwa Province, Pakistan was investigated as a complement to the Indian strain above. Initial assessments found that the strain from Pakistan can infect a significant number of *I. glandulifera* regional biotypes that are resistant to the Indian strain. Permission to release this additional strain was granted in January 2017 and, after conducting assessments of regional-biotype susceptibility, the most virulent strain of the rust was released at field sites during 2017.

Our goal is to provide practical, robust, rapid, and inexpensive methods for characterising differences between plant biotypes that could be used generally within the plant sciences and specifically to optimise the matching between a particular biological control agent and susceptible plant biotypes faster and cheaper than our current method (growing plants and then testing them empirically). Various techniques are available that may be deployed to meet this specific goal. Available analytical techniques include: DNA barcoding [14, 15]; next-generation sequencing [16], particularly reversible-terminator sequencing [17, 18] and nanopore sequencing [19, 20]; and proteome-analytical techniques [21], which have been comprehensively reviewed by Aslam et al. [22]. From this vast array of available analytical techniques, we have selected matrix-assisted laser-desorption and ionisation time-of-flight mass spectrometry (MALDI-TOF MS) because this technique is capable of discrimination between species [23, 24] as well as between regional biotypes within species [24], and also has the advantage of being both rapid and very inexpensive in terms of reagent usage and time required for sample processing [23].

Now a well-established laboratory technique, MALDI-TOF MS is a powerful tool for the analysis of protein-containing samples. Large proteins can be prepared intact in the gas phase carrying predominantly a single positive charge [25] by means of the MALDI soft ionisation process [26]. The time-of-flight of a charged protein along a tube held at high vacuum, after acceleration in an electrical field, is proportional to the square root of the mass-over-charge ratio for the protein [27]. As a consequence of this simple relationship, a mass spectrum can be generated from the time-of-flight values for such gas-phase and charged protein components in a particular biological sample [27]. For the characterisation and identification of protein-containing biological samples, the mass spectrum of a subset of the expressed proteome is generally employed (normally the highly-expressed acid-soluble proteins, including many ribosomal proteins) [28].

Human clinical microbiology, with a primary focus on the diagnosis of bacterial and yeast infections, has been a key driver behind the development of MALDI-TOF MS sample preparation and analysis [28], and this area has been reviewed extensively by Clark et al. [27], along with common methods used for MALDI-TOF MS sample preparation. Additional microbiologically-focussed methods have also been developed for use with yeasts [29], filamentous fungi [30–33], and mycobacteria [34]. As plant materials are not particularly well suited to many of the above methods [35], we have developed, in response, a highly-simplified and inexpensive method for sample preparation that has broad applicability to bacteria, fungi, insects, and plants [23]. This method lyses plant cells by maceration in aqueous acetonitrile containing trifluoroacetic acid (TFA) to selectively extract acid-soluble proteins, and lysis and extraction are carried out in the presence of near-saturated and inexpensive-grade MALDI matrix. The resulting matrix-saturated lysate, containing acid-solubilised proteins, is then simply dried down directly onto the MALDI-TOF MS sample plate and analysed.

We have previously used the above method to distinguish between closely-related *Impatiens* species and between regional biotypes of the invasive weed *I. glandulifera* using leaf samples [24]. Whilst there is no fundamental problem working with leaf material, one limitation is that fresh or promptly-frozen and frozen-stored leaf material is required for MALDI-TOF MS analysis [36, 37]. In order to overcome this limitation, Reeve and Buddie [36] have developed a simple and inexpensive method for the practical storage of field-sample proteins, dried down onto filter paper, for subsequent MALDI-TOF MS analysis. In this method, leaf biomass is crushed onto filter paper and dried and, if required, the dried and protein-impregnated filter paper can then be treated with (aqueous) alcohol, followed by drying, in order to inactivate potential microorganisms of concern [38]. After dry storage, proteins are extracted from the paper in acetonitrile/TFA/water/matrix as above. This method also has the advantage that dried protein, rather than viable plant or seed material (which may be subject to quarantine restriction for transfer across national borders, for example under EC directive 2000/29/EC) can be transported for subsequent MALDI-TOF MS analysis. A second limitation to working with leaf material is that high-level within-species discrimination is sensitive to the developmental stage of the sampled leaf, with newly emerging leaves giving less reliable spectra [24]. Given that younger leaves are actively growing, and that protein expression changes during plant aging [39], this is perhaps unsurprising. Whilst considering the above, we sought to develop additional MALDI-TOF MS sample-preparation methods that would be suitable for the analysis of seed material, which might possibly be more stable in storage compared to leaf material and/or less dependent upon the age of the sampled material. Additional motivations also included the opportunity to extend the applicability of MALDI-TOF MS to situations in which leaf material might not be readily available (for example, study of seeds separated from their parent plants, such as archived seed-repository material; seeds dispersed by wind and by animals, birds, or human activities such as farming or leisure; and also seed banks in soil, detritus, and on tools, boots, and farming equipment).

In the current paper, we describe the development of such a complementary MALDI-TOF MS-based method for use with seeds, and demonstrate its utility by successfully discriminating between the same four regional biotypes of *I. glandulifera* from within the UK, which differ in their susceptibility to the biological control agent *P. komarovii* var. *glanduliferae*.

## Materials and methods

### Seed collection and storage

Seeds of *I. glandulifera* were sourced directly from field populations in 2016 and 2017. Seeds were collected from four sites in the UK: Harmondsworth Moor, Middlesex; Silwood Park, Berkshire; Rhosmaen, Carmarthenshire; and Lampeter, Ceredigion. Seeds were collected from multiple plants across each site. Upon return to the laboratory, seeds were air dried at room temperature for 1 week before storage in the dark at 4 °C until extraction.

### MALDI-TOF-MS

Mass spectrometry covering the range 2 kDa to 20 kDa was carried out using a Bruker Microflex LT linear-mode instrument running the MALDI Biotyper 4.0 applications (Bruker Daltonik, Bremen, Germany), using a 60 Hz frequency and 3 ns pulse-duration nitrogen laser (70 µJ, with maximum output 225 µJ), with a wavelength of 337 nm and spot size of 100 µm, with 240 laser shots per sample. The laser settings were Global Attenuator Offset (0%), Attenuator Offset (20%), and Attenuator Range (30%), and the ion-source voltage was 19.98 kV. Bruker MBT Biotarget 96 plates (Bruker ref. 1840375) were used for all samples in this study. Calibration was carried out using the manufacturer’s ‘BTS’ controls (*E. coli* proteins supplemented with ribonuclease A and myoglobin), using peaks with masses at 3637.8; 5096.8; 5381.4; 6255.4; 7274.5; 10,300.2; 13,683.2, and 16,952.3 for calibration according to the manufacturer’s instructions. Spectra were acquired using MALDI Biotyper RTC Version 4.0 (Build 19) using the manufacturer’s standard settings (Centroid peak-detection algorithm and TopHat baseline subtraction). Database entries were made as single-spectra MSPs using the Bruker Online Client software suite (Version 4.0.19, Bruker Daltonik, Bremen, Germany) using the manufacturer’s standard settings. For spectral comparisons, Bruker identification scores were derived using the standard Bruker algorithm. This first converts raw mass spectra into peak lists, which are then compared between spectra. Three separate values are computed: the number of peaks in the reference spectrum that have a closely-matching partner in the test spectrum (value range 0–1), the number of peaks in the test spectrum that have a closely-matching partner in the reference spectrum (value range 0–1), and the peak-height symmetry of the matching peaks (value range 0–1). The above three values are multiplied together and normalised to 1000, and the base-10 logarithm is then taken to give the final Bruker score (range 0-3). Bruker scores of scores between 2.3 and 3.0 indicate very close relatedness, scores between 2.0 and 2.3 indicate close relatedness, and scores below 1.7 indicate low relatedness. Spectral comparisons using principal-component analysis (PCA) were also carried out using the Bruker On-line Client software. PC1 versus PC2 ordination plot were generated, PCA analysis was unsupervised, and all peaks were weighted equally.

### Reagents

≥ 99.8% ethanol, ≥ 98% (TLC-grade) α-cyano-4-hydroxycinnamic acid (HCCA) matrix, LC–MS-grade acetonitrile, and 99% ReagentPlus^®^-grade TFA were purchased from Sigma (Gillingham, UK). CHROMASOLV™ LC–MS-grade water was purchased from Fluka (Loughborough, UK).

### Sample preparation

For MALDI-TOF MS spectra of *I. glandulifera* acid-soluble seed proteins, single seeds were macerated in 100 µl of (11 mg/ml HCCA matrix in 65% (v/v) acetonitrile, 2.5% (v/v) TFA, and 32.5% (v/v) water) (referred to as Solution 1 in the following) using the blunt end of a plastic inoculating loop. Seed debris was pelleted by centrifugation at 14,100*g* for 1 min in a miniSpin^®^ plus centrifuge (Eppendorf, Stevenage, UK). One microlitre of the resulting supernatant (or dilutions thereof in Solution 1 as indicated) was then pipetted onto the Bruker sample plate, air dried, and loaded into the spectrometer.

## Results

Initial method-development trials macerated *I. glandulifera* seeds, collected in 2017, in 100 µl of Solution 1 (described in the methods section). Additional file 1: Figure S1 shows an example tube (Harmondsworth Moor reference sample 1) after extraction. Despite the significant amount of biomass extracted (clearly visible in Additional file 1: Figure S1), no spectra were obtained from 1 µl aliquots of acid-soluble seed proteins from the four regional biotypes (data not shown). Whilst observing the real-time data acquisition, it was apparent that very little ionic material was reaching the detector. The matrix crystals on the sample plate were also observed to have a dark, gelatinous, and wrinkled appearance rather than the whitish crystalline morphology normally associated with successful MALDI-TOF MS. Reasoning that the high concentration of material extracted from the seeds is perhaps inhibitory to the MALDI desorption and/or ionisation process, we therefore carried out serial dilutions of one of the above 100 µl extractions with the aim of reducing any such putative inhibitory material to an insignificant level before the concentration of acid-soluble proteins that could be used to characterise and/or identify the seeds fell too low to detect.

**Fig. 1:**
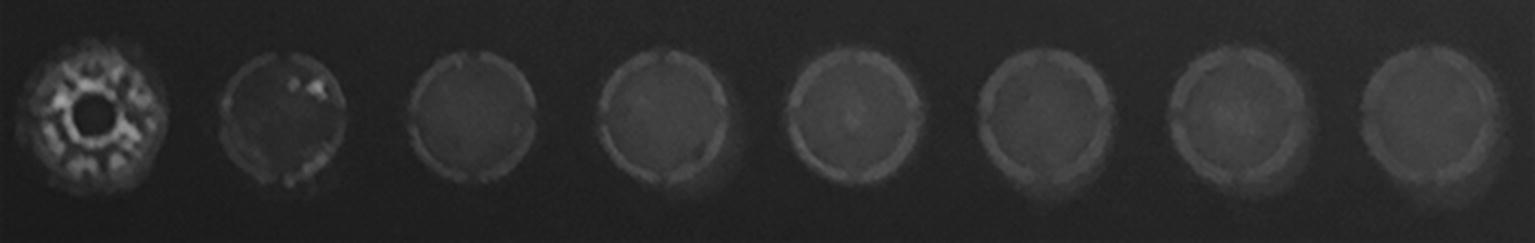
One microlitre aliquots, dried onto a Bruker MALDI-TOF MS sample plate, from 2-fold serial dilutions in Solution 1 of a single *I. glandulifera* seed (Harmondsworth Moor reference sample 1) initially extracted in 100 µl of Solution 1. The samples shown are, from left to right, undiluted, 2-fold dilution, 4-fold dilution, 8-fold dilution, 16-fold dilution, 32-fold dilution, 64-fold dilution, and 128-fold dilution

Figure 1 shows 1 µl aliquots, dried onto a Bruker MALDI-TOF MS sample plate, from 2-fold serial dilutions in Solution 1 of a single *I. glandulifera* seed (Harmondsworth Moor reference sample 1, as above) initially extracted in 100 µl of Solution 1.

**Fig. 2:**
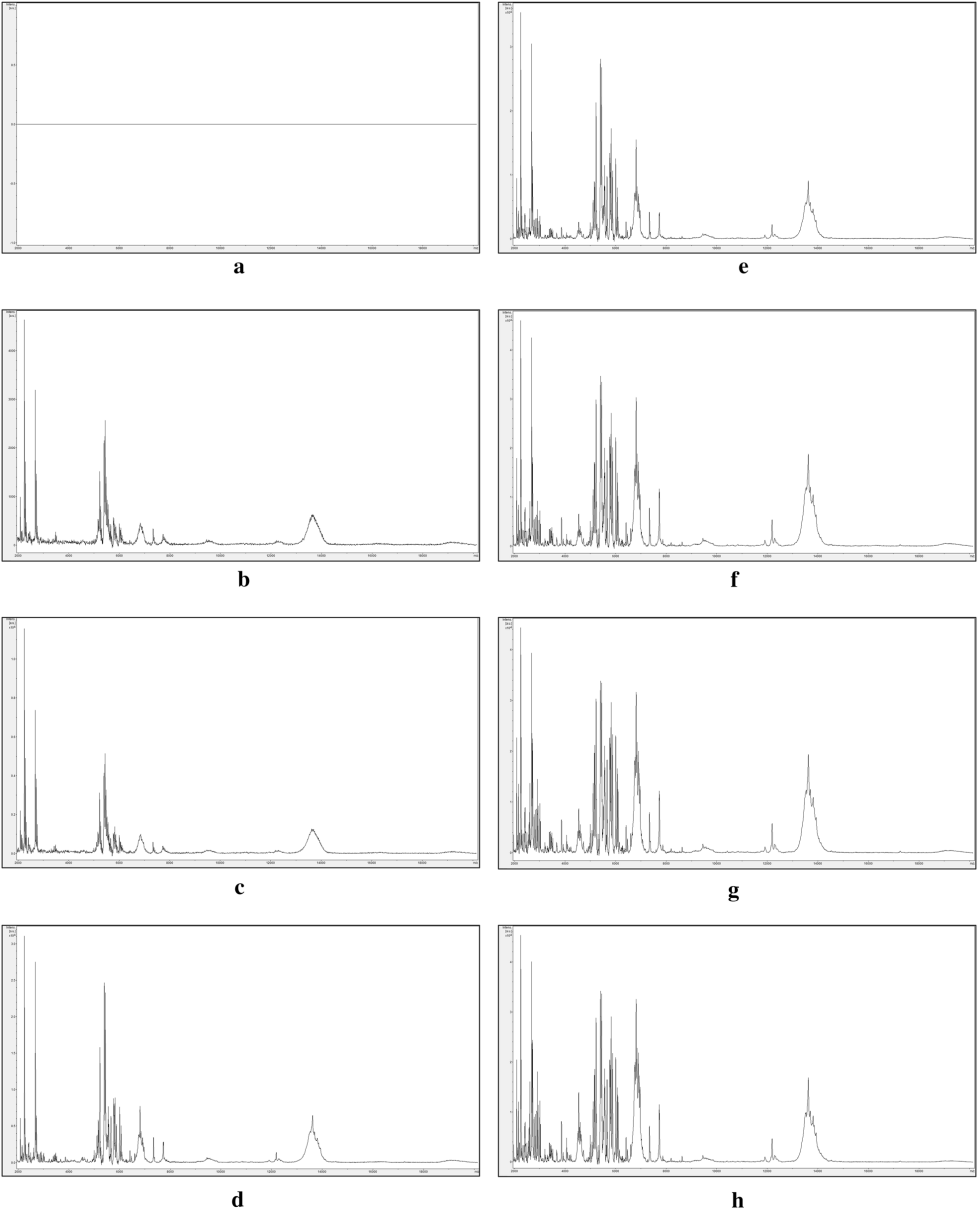
MALDI-TOF MS spectra of acid-soluble seed proteins from 2-fold serial dilutions in Solution 1 of a single *I. glandulifera* seed (Harmondsworth Moor reference sample 1) initially extracted in 100 µl of Solution 1. The samples shown are: **a** undiluted, **b** 2-fold dilution, **c** 4-fold dilution, **d** 8-fold dilution, **e** 16-fold dilution, **f** 32-fold dilution, **g** 64-fold dilution, and **h** 128-fold dilution. The y axes show arbitrary intensity units and the x axes show molecular weight. Spectra are shown baseline-subtracted, smoothed, y-axis-autoscaled, and covering the mass range 2 kDa to 20 kDa (with x-axis scale increments of 2 kDa)

Figure 1 showed that, as the dilution of the 100 µl extract increased, the matrix-crystal morphology gradually transitioned from dark, gelatinous, and wrinkled (undiluted), through dark and gelatinous (2-fold and 4-fold dilution), to the whitish crystalline morphology normally associated with successful MALDI-TOF MS (8-fold dilution to 128-fold dilution). Figure 2 shows the MALDI-TOF MS spectra over the mass range 2 kDa to 20 kDa obtained from the matrix crystals shown in Fig. 1.

**Fig. 3:**
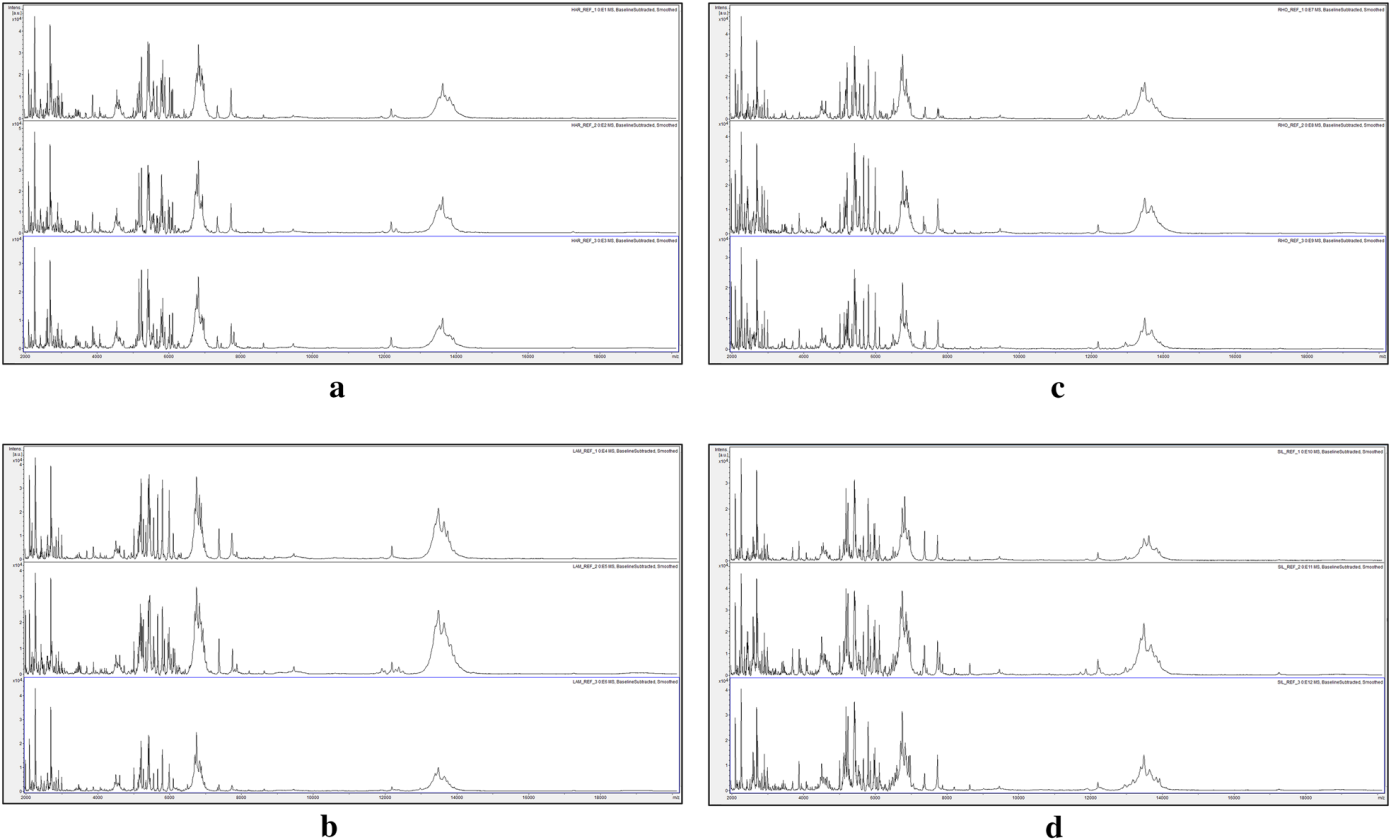
Triplicate reference-sample MALDI-TOF MS spectra of acid-soluble seed proteins from randomly-selected seeds collected in 2017 from **a** Harmondsworth Moor, **b** Lampeter, **c** Rhosmaen, and **d** Silwood Park. The y axes show arbitrary intensity units and the x axes show molecular weight. Spectra are shown baseline-subtracted, smoothed, y-axis-autoscaled, and covering the mass range 2 kDa to 20 kDa (with x-axis scale increments of 2 kDa)

Figure 2 showed that, as the dilution of the original 100 µl extract increased, the MALDI-TOF MS spectra gradually transitioned from no spectrum obtained (undiluted), through a relatively small number of peaks obtained (2-fold to 8-fold dilution), to a greater number of peaks obtained (16-fold dilution to 128-fold dilution), with slightly increasing peak richness and resolution with increasing dilution through to 128-fold dilution in Solution 1. On the basis of the above serial dilution data, we therefore opted for a method based upon 100 µl initial extraction of single *I. glandulifera* seeds in Solution 1 followed by a further 100-fold dilution in Solution 1 before drying 1 µl aliquots onto the sample plate for MALDI-TOF MS analysis. Using this method, we first derived reference spectra, from seeds collected in 2017, for four UK regional biotypes of *I. glandulifera* (from Harmondsworth Moor, Lampeter, Rhosmaen, and Silwood Park—regional biotypes that differ in their susceptibility to the biological control agent *P. komarovii* var. *glanduliferae*). The triplicate reference spectra obtained (each from a different seed randomly selected from seeds gathered at each geographical site) are shown in Fig. 3.

Figure 3 showed that, using the method developed and described above, MALDI-TOF MS spectra of acid-soluble seed proteins from all four regional biotypes were obtained that showed good peak richness and high replicate-to-replicate reproducibility. Broad peak complexes were observed centred around 6.8 kDa and 13.5 kDa but most of the remaining peaks were not significantly broadened.

**Fig. 4:**
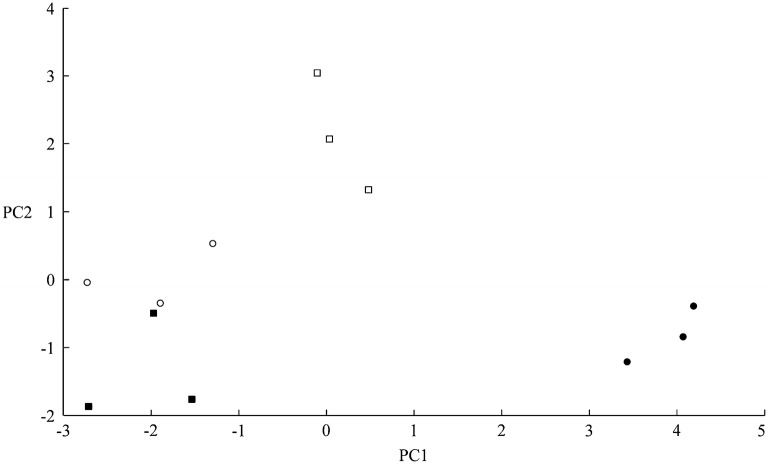
PCA ordination plot for the triplicate reference-sample MALDI-TOF MS spectra of acid-soluble seed proteins from seeds collected in 2017 from (filled circle) Harmondsworth Moor, (filled square) Lampeter, (open circle) Rhosmaen, and (open square) Silwood Park. PCA analysis was unsupervised, and all peaks were weighted equally

Having achieved our primary goal of providing a practical and robust method for generating high-quality MALDI-TOF MS spectra from seeds, we investigated whether this could be used for discrimination between the above regional biotypes (which is a test of more than just the MALDI-TOF MS sample-preparation method, requiring that the sum of all experimental and within-biotype variance is lower than the variance between regional biotypes). As a preliminary visual analysis, the spectra shown in Fig. 3 were used for principal-component analysis (PCA) as described in the methods section. An ordination plot for these 12 reference-sample MALDI-TOF MS spectra is shown in Fig. 4.

Figure 4 showed well-separated grouping of the reference-sample spectra obtained from Harmondsworth Moor (filled circle) and Silwood Park (open square) but less-well-separated groupings of the reference-sample spectra obtained from Lampeter (filled square) and Rhosmaen (open circle). On the basis of the above PCA data, we therefore opted to test whether we could identify single *I. glandulifera* seeds using a much more stringent method: blind testing of test-seed spectra against the above 12 reference spectra. Table 1 shows the results for 12 seeds collected in 2017 (three each from Harmondsworth Moor, Lampeter, Rhosmaen, and Silwood Park) that were analysed blind and for which their MALDI-TOF MS spectra were compared to all twelve reference spectra from seeds also collected in 2017, calculating the average Bruker score against the triplicate reference spectra for seeds from Harmondsworth Moor, Lampeter, Rhosmaen, and Silwood Park.

**Table 1:** Average Bruker score against triplicate reference spectra from seeds collected in 2017 for 12 blind-test seeds also collected in 2017 from Harmondsworth Moor, Lampeter, Rhosmaen, and Silwood Park (three seeds from each site)

Blind-test number	Average Bruker score against triplicate reference spectra for seeds from				Identification call	Accuracy after unblinding
	Harmondsworth Moor	Lampeter	Rhosmaen	Silwood Park		
1	1.507	2.357	2.154	1.742	Lampeter	Correct
2	1.312	2.111	2.305	1.790	Rhosmaen	Correct
3	1.473	1.778	1.801	2.419	Silwood Park	Correct
4	1.317	1.993	2.306	1.821	Rhosmaen	Correct
5	2.132	1.465	1.452	1.476	Harmondsworth Moor	Correct
6	2.155	1.495	1.429	1.412	Harmondsworth Moor	Correct
7	1.185	2.196	2.175	1.541	Lampeter	Correct
8	1.403	1.805	1.886	2.252	Silwood Park	Correct
9	1.537	2.257	2.383	1.892	Rhosmaen	Correct
10	1.193	1.404	1.804	2.422	Silwood Park	Correct
11	0.969	2.261	2.089	1.900	Lampeter	Correct
12	2.384	1.124	1.574	1.542	Harmondsworth Moor	Correct

The highest average Bruker score is given as the identification call in the penultimate column, and the accuracy of this after unblinding the data is shown the final column

**Fig. 5:**
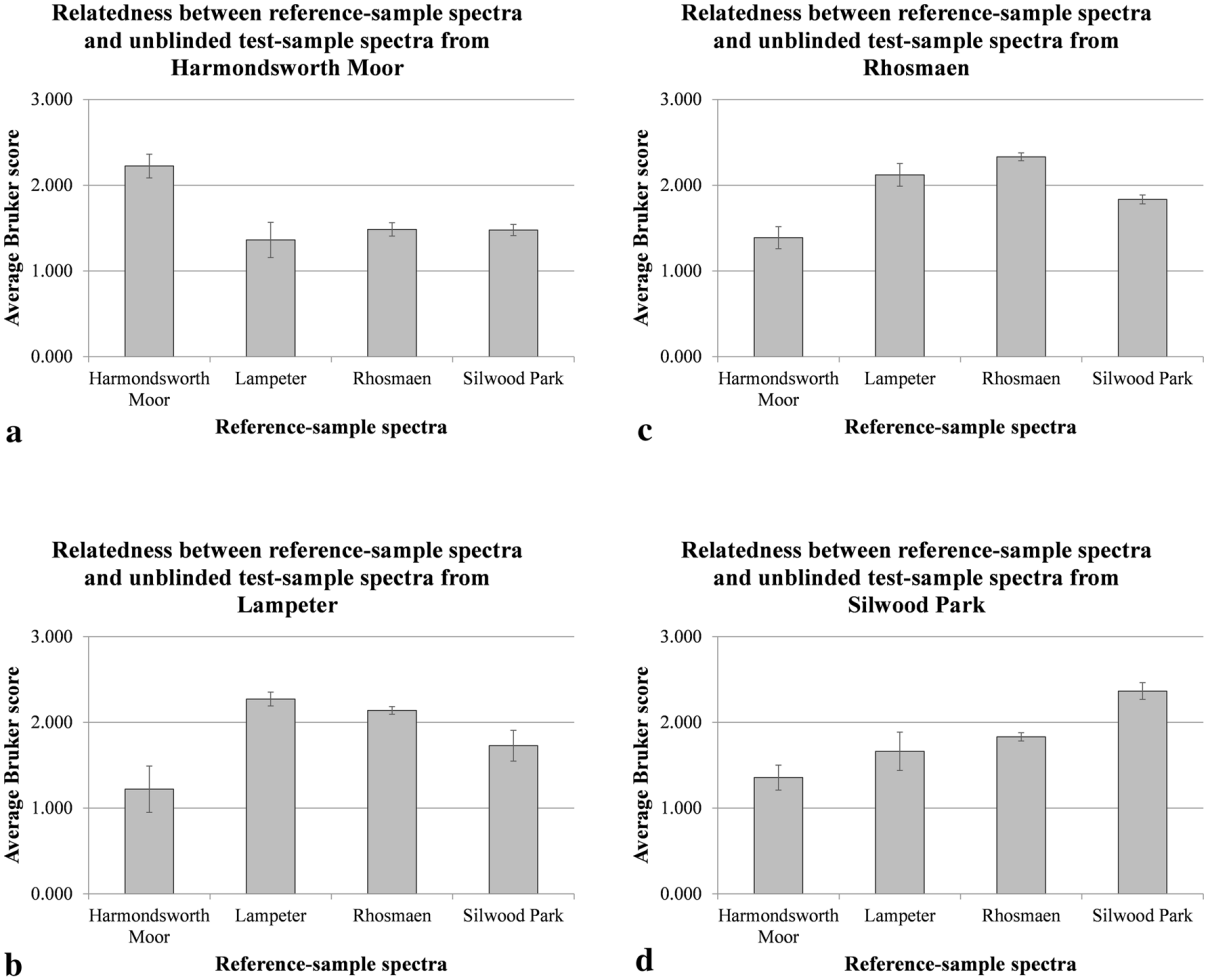
Average values, for each site-specific set of three unblinded test samples after Table 1, of the average Bruker scores against the site-specific triplicate reference spectra, with error bars indicating one standard deviation either side of the mean

Table 1 showed that the identification calls for all 12 blind-tested seeds were correct after unblinding the data. Knowing the identity of the above 12 test samples after unblinding the data, we were also able to calculate average values, for each site-specific set of three test samples, of the average Bruker scores against the site-specific triplicate reference spectra. These average values (Additional file 1: Table S1), with error bars indicating one standard deviation either side of the mean (Additional file 1: Table S2), are shown in Fig. 5.

Figure 5 showed that the unblinded test-sample spectra from Harmondsworth Moor are most closely related to the reference-sample spectra from Harmondsworth Moor, with clear separation from the remaining reference-sample spectra. Of the non-cognate reference-sample-spectra, the unblinded test-sample spectra from Harmondsworth Moor were most closely related to the reference-sample spectra from Rhosmaen, followed by Silwood Park, and then Lampeter. The unblinded test-sample spectra from Lampeter were most closely related to the reference-sample spectra from Lampeter, with separation from the remaining reference-sample spectra. Of the non-cognate reference-sample-spectra, the unblinded test-sample spectra from Lampeter were most closely related to the reference-sample spectra from Rhosmaen, followed by Silwood Park, and then Harmondsworth Moor. The unblinded test-sample spectra from Rhosmaen were most closely related to the reference-sample spectra from Rhosmaen, with separation from the remaining reference-sample spectra. Of the non-cognate reference-sample-spectra, the unblinded test-sample spectra from Rhosmaen were most closely related to the reference-sample spectra from Lampeter, followed by Silwood Park, and then Harmondsworth Moor. The unblinded test-sample spectra from Silwood Park were most closely related to the reference-sample spectra from Silwood Park, with clear separation from the remaining reference-sample spectra. Of the non-cognate reference-sample-spectra, the unblinded test-sample spectra from Silwood Park were most closely related to the reference-sample spectra from Rhosmaen, followed by Lampeter, and then Harmondsworth Moor.

As a further and more-demanding test for our method, we also opted to test whether we could identify single *I. glandulifera* seeds by blind testing of test-seed spectra from seeds collected in one year against reference spectra from seeds collected in a different year. Table 2 shows the results for 12 seeds collected in 2016 (three each from Harmondsworth Moor, Lampeter, Rhosmaen, and Silwood Park) that were analysed blind and for which their MALDI-TOF MS spectra were compared to all twelve of the above reference spectra from seeds collected in 2017, again calculating the average Bruker score against the triplicate reference spectra for seeds from Harmondsworth Moor, Lampeter, Rhosmaen, and Silwood Park.

**Table 2:** Average Bruker score against triplicate reference spectra from seeds collected in 2017 for 12 blind-test seeds collected in 2016 from Harmondsworth Moor, Lampeter, Rhosmaen, and Silwood Park (three seeds from each site)

Blind-test number	Average Bruker score against triplicate reference spectra for seeds from				Identification call	Accuracy after unblinding
	Harmondsworth Moor	Lampeter	Rhosmaen	Silwood Park		
13	1.455	2.170	2.115	1.842	Lampeter	Correct
14	0.972	2.247	2.165	1.949	Lampeter	Incorrect (Rhosmaen)
15	2.322	1.390	1.378	1.251	Harmondsworth Moor	Correct
16	1.155	2.014	2.026	1.639	Rhosmaen	Correct
17	2.041	1.525	1.487	1.603	Harmondsworth Moor	Correct
18	1.384	1.433	1.650	2.399	Silwood Park	Correct
19	1.325	2.207	2.181	1.645	Lampeter	Correct
20	1.404	1.495	1.621	2.336	Silwood Park	Correct
21	1.710	1.855	1.996	2.350	Silwood Park	Correct
22	1.136	2.185	1.936	1.772	Lampeter	Correct
23	2.006	1.754	1.734	1.587	Harmondsworth Moor	Correct
24	1.488	2.280	2.371	1.779	Rhosmaen	Correct

The highest average Bruker score is given as the identification call in the penultimate column, and the accuracy of this after unblinding the data is shown the final column

Table 2 showed that the identification calls for 11 out of 12 blind-tested seeds were correct after unblinding the data. For identification into three categories (Harmondsworth Moor, Lampeter/Rhosmaen, and Silwood Park), our method was still robust, even using reference sample spectra collected during a different year to the seeds tested. For a more-stringent discrimination between Lampeter and Rhosmaen, our method was more reliable when using test and reference seeds collected in the same year.

## Discussion

Invasive weeds are a worldwide and significant economic problem, for which biological control using co-evolved natural enemies isolated from the region of origin of the target species can be an environmentally-sustainable and effective solution. Our focus in the current paper has been *I. glandulifera*, an invasive annual weed native to the foothills of the Himalayas, for which two strains of the rust-based biological control agent *P. komarovii* var. *glanduliferae* are currently available (one originating from India and the other from Pakistan). Experience from past weed-biocontrol programmes, particularly those using plant pathogens that are often intrinsically linked to their hosts, suggests that the susceptibility of some weed populations to their biocontrol agent can vary [40, 41]. Current practice for optimising the pairing between biological control agent strains and target-weed regional (or other) biotypes is largely empirical (target plants obtained from a particular site of interest are grown and tested for their susceptibility to the available biological control agent strains), which can be a slow and a relatively-expensive process. As an alternative, we have investigated MALDI-TOF MS as a means of discriminating between regional biotypes with known biological control agent susceptibility profiles principally because this method is both rapid and very inexpensive in terms of reagent usage and time required for sample processing.

For the analysis of plant seeds in this paper, we have employed a method for MALDI-TOF MS sample preparation that uses acetonitrile containing TFA to selectively extract acid-soluble proteins, with extraction also carried out in the presence of near-saturated and inexpensive-grade MALDI matrix (Solution 1 as described in the methods section). As a significantly-stronger acid than formic acid (frequently employed in MALDI-TOF MS sample-preparation methods [27]), TFA was chosen for acidification in order to give comparable proton concentrations from much lower concentrations of acid in order to reduce significantly the amount of odorous material evaporating from the reagents and sample plate during use. We also chose to premix the extracted proteins and MALDI matrix, and to dry these down together. Finally, we used inexpensive-grade MALDI matrix in order to make our method economically practical, using C2020-10G HCCA matrix supplied by Sigma (which is around 500 times cheaper per gram than many 99%-pure ‘MALDI-grade’ reagents). In terms of time taken and costs, our MALDI-TOF MS takes around 2 min of labour time per sample, and has reagent costs of 1.2 UK pence per sample (0.8 UK pence for acetonitrile, 0.1 UK pence for TFA, and 0.3 UK pence for HCCA matrix) [23].

In order to test the discriminating power of our method, we chose to work with seeds from four UK regional biotypes of *I. glandulifera* that we have previously demonstrated discrimination between using leaf samples [24]. These four regional biotypes (originating from Harmondsworth Moor, Lampeter, Rhosmaen, and Silwood Park) also differ in their susceptibility to the biological control agent *P. komarovii* var. *glanduliferae*. As a rigorous and stringent test for our method, we opted for blind testing of test-seed spectra against a panel of four triplicate reference spectra of acid-soluble seed proteins from seeds obtained from each geographical site, using seeds all collected during the same year. With a one in 16,777,216 probability of obtaining by chance the observed correct identification in 12 consecutive blind tests, each with a one in four probability of being correct by chance, this blind testing confirmed that our method could reliably discriminate between randomly-selected *I. glandulifera* seeds originating from these four UK regional biotypes. As evidenced by the PCA ordination plot generated from the reference-sample spectra (Fig. 4), the *I. glandulifera* regional biotypes from Harmondsworth Moor and Silwood Park were readily discriminable through analysis of the MALDI-TOF MS spectra of their acid-soluble proteins but the regional biotypes from Lampeter and Rhosmaen were rather similar and were not discriminable through PCA. Using the more stringent blind-testing method above, it was just possible to discriminate between the Lampeter and Rhosmaen regional biotypes without error-bar overlap at one standard deviation either side of the mean. The spectral-comparison data in Fig. 5 also supported the PCA ordination plot further indicating closeness between the Lampeter and Rhosmaen samples, with the Harmondsworth Moor and Silwood Park samples again being much more clearly discriminable. As a further test for our method, we also tested whether we could identify single *I. glandulifera* seeds by blind testing of test-seed spectra from seeds collected in one year against reference spectra from seeds collected in the previous year. This is a more-demanding test for our method, especially given the closeness of the Lampeter and Rhosmaen spectra. As we carried out 11 successful blind tests, each with a one in four probability of being correct by chance, the probability of achieving the successful identifications shown in Table 2 by chance is still one in 4,194,304. For identification into three categories (Harmondsworth Moor, Lampeter/Rhosmaen, and Silwood Park), our method was still robust, even using reference sample spectra collected during a different year to the seeds tested. For a more-stringent discrimination between Lampeter and Rhosmaen, however, our method was more reliable when using test and reference seeds collected in the same year.

As mentioned above, when discriminating between regional biotypes of *I. glandulifera* using leaf material [23], we observed that more-reliable results were obtainable from mature leaves than from newly-developing leaves, which imposes a minor limitation when working with leaf material. Analysis of seed material, by comparison, may possibly be less constrained in terms of sample age, and may therefore offer a valuable complementary method for discriminating between plant species and between regional biotypes within a species. Additionally, it has been demonstrated that seeds can be stored in the fridge for a number of years and still give reliable spectra. Successful discrimination between invasive-weed regional biotypes using MALDI-TOF MS-based analysis of seeds as demonstrated above raises the intriguing possibility that further deployment of this method might enable the identification of *I*. *glandulifera* regional biotypes to a level that could predict their susceptibility to available strains of *P. komarovii* var. *glanduliferae.* This could significantly increase both the efficiency and efficacy of biological control of this invasive weed—a possibility that we intend to pursue in future studies.

In addition to application in the area of biological control, the method described above extends the applicability of MALDI-TOF MS to situations in which leaf material might not be readily available. Seeds separated from their parent plants may now be amenable to MALDI-TOF MS analysis (for example, archived seed-repository material; seeds dispersed by wind and by animals, birds, or human activities such as farming or leisure; and also seed banks in soil, detritus, and on tools, boots, and farming equipment).

## Conclusions

We have successfully developed a practical, robust, rapid, and inexpensive method that generates peak-rich and highly-reproducible MALDI-TOF MS spectra of acid-soluble *I. glandulifera* seed proteins. Employing a combination of principal-component analysis and blind-tested comparison between reference-sample MALDI-TOF MS spectra and test-sample spectra, we have been able to discriminate, on the basis of the acid-soluble seed-protein spectra generated by our method, between four regional biotypes of *I. glandulifera* from within the UK that differ in their susceptibility to the biological control agent Himalayan balsam rust (*P. komarovii* var. *glanduliferae*). MALDI-TOF MS-based analysis of seed material therefore has the potential to improve the efficiency and efficacy of weed biological control using co-evolved natural enemies of invasive non-native plant species, through the optimal matching of biological control agents with susceptible regional biotypes.

## Additional file

**Additional file 1: Figure S1.** An example 1.5 ml Eppendorf tube (Harmondsworth Moor reference sample 1) showing the significant amount of biomass extracted from a single I. glandulifera seed in 100 µl of Solution 1. **Table S1.** Average values, for each site-specific set of three unblinded test samples after Table 1, of the average Bruker scores against the site-specific triplicate reference spectra. **Table S2.** Standard deviations, for each site-specific set of three unblinded test samples after Table 1, of the average Bruker scores against the site-specific triplicate reference spectra.

## Abbreviations

MALDI-TOF MS: matrix-assisted laser-desorption and ionisation time-of-flight mass spectrometry
HCCA: α-cyano-4-hydroxycinnamic acid
TFA: trifluoroacetic acid
PCA: principal-component analysis
