# Supplementary material for: Discrimination between regional biotypes of Impatiens glandulifera using a simple MALDI-TOF MS-based method for use with seeds
Source: Plant Methods. 2019 Mar 14;15:25. doi: 10.1186/s13007-019-0412-1 (PMC6416845; doi:10.1186/s13007-019-0412-1)
Supplement: Supplementary file 1 — Additional file 1: Figure S1. An example 1.5 ml Eppendorf tube (Harmondsworth Moor reference sample 1) showing the significant amount of biomass extracted from a single I. glandulifera seed in 100 µl of Solution 1. Table S1. Average values, for each site-specific set of three unblinded test samples after Table 1, of the average Bruker scores against the site-specific triplicate reference spectra. Table S2. Standard deviations, for each site-specific set of three unblinded test samples after Table 1, of the average Bruker scores against the site-specific triplicate reference spectra. [file 13007_2019_412_MOESM1_ESM.docx]

**Figure S1**

**Figure S1** An example 1.5 ml Eppendorf tube (Harmondsworth Moor reference sample 1) showing the significant amount of biomass extracted from a single *I. glandulifera* seed in 100 µl of Solution 1.

**Table S1**

|  |  | Reference samples | | | |
| --- | --- | --- | --- | --- | --- |
|  |  | Harmondsworth Moor | Lampeter | Rhosmaen | Silwood Park |
| Test samples | Harmondsworth Moor | 2.224 | 1.361 | 1.485 | 1.477 |
|  | Lampeter | 1.220 | 2.271 | 2.139 | 1.728 |
|  | Rhosmaen | 1.389 | 2.120 | 2.331 | 1.834 |
|  | Silwood Park | 1.356 | 1.662 | 1.830 | 2.364 |

**Table S1** Average values, for each site-specific set of three unblinded test samples after Table 1, of the average Bruker scores against the site-specific triplicate reference spectra.

**Table S2**

|  |  | Reference samples | | | |
| --- | --- | --- | --- | --- | --- |
|  |  | Harmondsworth Moor | Lampeter | Rhosmaen | Silwood Park |
| Test samples | Harmondsworth Moor | 0.139 | 0.206 | 0.078 | 0.065 |
|  | Lampeter | 0.271 | 0.081 | 0.045 | 0.180 |
|  | Rhosmaen | 0.128 | 0.132 | 0.045 | 0.052 |
|  | Silwood Park | 0.146 | 0.224 | 0.048 | 0.097 |

**Table S2** Standard deviations, for each site-specific set of three unblinded test samples after Table 1, of the average Bruker scores against the site-specific triplicate reference spectra.
